# The impact of cell phone use after light out on sleep quality, headache, tiredness, and distractibility among high school students: Cross sectional study

**DOI:** 10.1016/j.heliyon.2025.e42655

**Published:** 2025-02-14

**Authors:** Hanan Alrubaia, Zainab Alabdi, Mohammed Alnaim, Nurah Alkhteeb, Abdulmohsen Almulhim, Ammar Alsalem, Rimas Aldarwish, Ibtisam Algouf, Abdullah Almaqhawi

**Affiliations:** a College of Medicine, King Faisal University, Al-Ahsa, Saudi Arabia; b King Fahad Hospital, Ministry of Health, Hofuf, Saudi Arabia; c Department of Family Medicine, Ministry of National Guard Health Affairs, Saudi Arabia; d Department of Family and Community Medicine, College of Medicine, King Faisal University, Al-Ahsa, Saudi Arabia

**Keywords:** Cell phones, Sleep quality, PSQI, Symptoms, High school students

## Abstract

To assess the impact of cell phone use after light out on sleep quality, headache, tiredness, and distractibility among high school students in the Eastern Province of Saudi Arabia. This cross-sectional study was conducted among high school students aged between 15 and 18 years old in the Eastern Province of Saudi Arabia. A self-administered questionnaire was distributed among students using a Google Form survey. The questionnaire includes socio-demographic characteristics, symptoms that affected personal or school life quality, and the Pittsburgh Sleep Quality Index (PSQI) to assess sleep quality. Of the 297 high school students, 71.8 % were females, and 54.2 % were 17–18 years old. The most common symptoms influencing personal or school quality of life are tiredness, insomnia, and energy loss. The prevalence of poor sleep quality was 73.4 %. The older age group and female gender were more likely to complain of poor sleep quality. A multivariate regression model identified headache and insomnia as the significant independent risk factors for poor sleep quality. This study determined that using cell phones after light out for messaging and calling was associated with various forms of sleep disorders. In addition, high school students with poor sleep quality tended to experience symptoms such as headaches, insomnia, loss of energy, distractibility, and tiredness. More longitudinal studies are needed to establish the effect of cell phone use after light out on sleep quality and other related symptoms that affect personal or school life among high school students.

## 1 Introduction

In the contemporary technological landscape, mobile phones and internet access have become indispensable societal elements. Their pervasive use across diverse demographics necessitates a comprehensive exploration of potential health implications. Notably, the practice of engaging with mobile phones (e.g., calls, texts) after lights out is particularly prevalent amongst adolescents and young adults. This behavior warrants specific attention due to its potential disruption of crucial physiological processes, including sleep cycles [1]. Mobile phones captivate the youth as they provide a sense of independence, identity, and reliability [1,2]. Furthermore, it is simply a source of amusement and allows them to stay continually connected with their friends and peers [1,3,4]. It seems that since interaction with peers is a common trait among the youth of all cultures, the propensity to use mobile phones is quite similar among adolescents and young adults across different cultures [1].

The use of cell phones after light-out negatively impacts various aspects of adolescents' lives. It reduces the quality and quantity of sleep, leading to sleep disturbances and poor sleep quality [5]. This can result in various issues such as headache, tiredness, and distractibility during the day. The use of social media on cell phones is also associated with poorer sleep quality and higher rates of depression among adolescents [5,6]. A study conducted in Makkah, Saudi Arabis, showed that cell phone addiction was high among school students, which was significantly associated with poor sleep quality [7]. Late-night socialising and extended screen time can disrupt high school students' natural sleep-wake cycle [8,9].

Inadequate or irregular sleep has been linked to a variety of health issues in high school students, including obesity, metabolic disorders, and mental health problems [10]. Addressing these sociocultural factors may be essential in formulating effective interventions to alleviate the health consequences of cell phone usage.

In this study, we aim to assess the impact of cell phone use after light out on sleep quality, headache, tiredness, and distractibility among high school students in the Eastern Province of Saudi Arabia.

## 2 Methods

### 2.1 Study design

This cross-sectional study used an online questionnaire to collect data on sleep disorders and cell phone use from parents in the Eastern region of Saudi Arabia on behalf of their high school children aged 15–18 years. The study included students within the specified age range whose parents were willing to provide informed consent for their participation.

### 2.2 Participants

Participants were required to reside in the Eastern region of Saudi Arabia. Students whose parents were unable or unwilling to provide informed consent, as well as those residing outside the Eastern region, were excluded from the study.

The survey link was distributed using social media platforms (X, Facebook, and WhatsApp) and by trained data collectors from various cities to gather the maximum number of participants from October 13, 2023, to January 16, 2024. This reliance on social media platforms may interdoce selection bias. Namely, students who are using these platforms would in theory be using their mobile phones more. Saudi Arabian parents were proxies responding on behalf of their children to fill out an anonymous survey.

#### 2.2.1 Sample size

According to the computer software (EPI INFO) sample size calculator, the minimal sample size was 305 with a confidence level of 95 % (sample size = Z2(p)(1-p)/c2). This study unfortunately have not met this sample size (297/305) which may further increase the margin of error.

#### 2.2.2 Questionnaire criteria

Parents answered a validated survey on sociodemographic data, cell phone usage, and sleep quality in vaArabic [11]. The questionnaire included a section for giving informed consent and protecting the participants' privacy. The reliability of the questionnaire was verified using consultants' reviews and a pilot study of the first ten responses using Cronbach's alpha test. The questionnaire consisted of 19 close-ended questions divided into three sections. The first section was designed to collect sociodemographic data about the students and their families.

The second section focused on the use of mobile phones after turning off the lights at bedtime, including the type of use such as receiving and sending text messages, and receiving and making calls. It also inquired about the frequency of use over the past month, with options ranging from every night to never. Additionally, this section examined the impact of using mobile phones after turning off the lights on headaches, insomnia, loss of energy, fatigue, and distraction among students, asking whether these symptoms had significantly affected the quality of their personal and/or school life over the past month. The third section represented the Pittsburgh Sleep Quality Index (PSQI), created by Carpenter & Andrykowski (1998) [12]. Parents were asked to rate their children's sleep quality, including duration, disturbances, latency, and efficiency over the previous month.

High school students' sleep disorders have been measured using the Pittsburgh Sleep Quality Index (PSQI). The PSQI is a 19-item self-rated questionnaire for assessing subjective sleep quality over the previous month. The 19 questions are subdivided into 7 domain component scores, and each is weighted equally from 0 to 3. The 7 domain scores are added to obtain a global PSQI score ranging from 0 to 21, with higher scores suggesting worse sleep quality. A cutoff point of 5 points was used for identifying cases of sleep disorder [13]. The different symptoms that significantly affected personal/school life quality due to cellphone use have been re-categorized for comparison. Categories such as "strongly disagree/disagree/neutral" were merged and classified as "no," and "agree/strongly agree" were merged and classified as "yes."

### 2.3 Ethical considerations

The study protocol was evaluated and approved by the Institutional Review Board at King Faisal University, Al-Ahsa, Saudi Arabia (Ethics Consent No. KFU-REC-2023-SEP-ETHICS1194, and informed consent was obtained from each participant before enrollment in the study. Confidentiality of the participants' responses was ensured, and the study adhered to relevant ethical guidelines.

### 2.4 Statistical analysis

Categorical variables were described as counts and proportions (%), while continuous variables were computed and expressed as mean and standard deviation. The relationship between sleep quality among the socio-demographic characteristics and the symptoms that affected the quality of personal or school life has been conducted using the Chi-square test. Significant results were then placed into a multivariate regression model to determine the significant independent risk factors for poor sleep quality. A p-value of less than 0.05 was considered statistically significant. All statistical data were analyzed using Statistical Packages for Social Sciences (SPSS) version 26 (Armonk, NY: IBM Corp., USA.).

## 3 Results

This study recruited two hundred and ninety-seven out of three hundred and five high school students (response rate: 97.4 %). As seen in Table 1, 54.2% were between 17 and 18 years old, with females being dominant (71.7 %). Most students were enrolled in public schools (74.4 %) and reside in Al-Ahsa (80.5 %). A high proportion of high school students indicated that they use cellphones at the light out to receive text messages (85.2 %) and use them every night (67.3 %).

**Table 1:** Socio-demographic characteristics of the high school students ^(n = 297)^.

Study variables	N (%)
Age group	
• 15–16 years	136 (45.8 %)
• 17–18 years	161 (54.2 %)
Gender	
• Male	84 (28.3 %)
• Female	213 (71.7 %)
School sector	
• Public school	221 (74.4 %)
• Private school	76 (25.6 %)
Residence location	
• Al-Ahsa	239 (80.5 %)
• Dammam	09 (03.0 %)
• Al-Khobar	13 (04.4 %)
• Al-Qatif	29 (09.8 %)
• Jubail	03 (01.0 %)
• Dhahran	04 (01.3 %)
Type of cellphone used at the light out^a^	
• Receiving text messages	253 (85.2 %)
• Sending text messages	191 (64.3 %)
• Receiving phone calls	92 (31.0 %)
• Calling	61 (20.5 %)
During the past month, the number of times used	
• Every night	200 (67.3 %)
• More than once a week	68 (22.9 %)
• Once a week	16 (05.4 %)
• 1–3 Times a month	06 (02.0 %)
• Never	07 (02.4 %)

a Variable with multiple response answers.

**Fig. 1:**
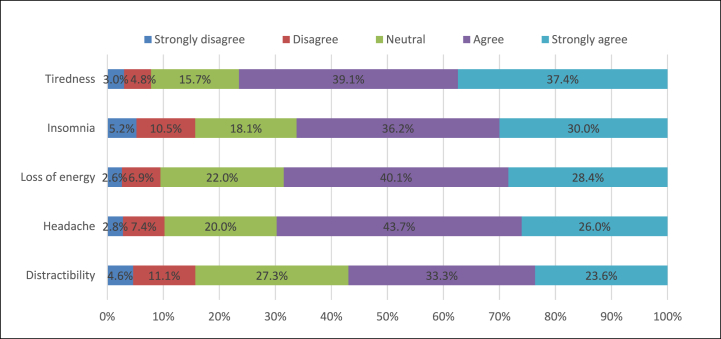
Symptoms that significantly affected the quality of personal/school life among cellphone users.

Fig. 1 depicts the symptoms that significantly affected personal/school life quality due to using cell phones. It can be observed that the most common symptoms that affected students' school life were tiredness (strongly agree: 37.4 %), followed by insomnia (strongly agree: 30 %), and loss of energy (strongly agree: 28.4 %).

Regarding the prevalence of sleep disorders (Table 2), using the PSQI questionnaire, it was revealed that the sleep latency domain showed the highest mean score (mean score: 1.44), followed by daytime dysfunction (mean score: 1:38) and sleep medication (mean score: 1.29). The global mean PSQI score was 7.89 (SD 3.64), with 73.4 % having poor sleep quality and the rest having good (26.6 %).

**Table 2:** Prevalence of sleep disorders using PSQI ^(n = 297)^.

PSQI Domains	Mean ± SD
Subjective sleep quality score	1.15 ± 0.83
Sleep latency score	1.44 ± 1.03
Sleep duration score	1.18 ± 1.05
Sleep efficiency score	1.04 ± 1.19
Sleep medication score	1.29 ± 0.67
Daytime dysfunction score	1.38 ± 0.92
**Global PSQI score**	**7.89 ± 3.64**

Level of sleep quality	**N (%)**
Poor (score >5)	218 (73.4 %)
Good (score ≤5)	79 (26.6 %)

When exploring the relationship between sleep quality according to the socio-demographic characteristics and the symptoms that affect the quality of personal or school life (Table 3), it was observed that students with poor sleep quality were more likely to be older (p = 0.002), female gender (p = 0.012), experienced symptoms such as headache (p < 0.001), insomnia (p < 0.001), losing energy (p < 0.001), distractibility (p < 0.001) and tiredness (p < 0.001).

**Table 3:** Relationship between sleep quality among the Socio-demographic characteristics and the symptoms that affected the quality of personal or school life ^(n = 297)^.

Factor	Sleep Quality		P-value^b^
	Poor N (%)^(n = 218)^	Good N (%)^(n = 79)^	
Age group			
• 15–16 years	88 (40.4 %)	48 (60.8 %)	**0.002 ∗∗**
• 17–18 years	130 (59.6 %)	31 (39.2 %)	
Gender			
• Male	53 (24.3 %)	31 (39.2 %)	**0.012 ∗∗**
• Female	165 (75.7 %)	48 (60.8 %)	
School sector			
• Public school	162 (74.3 %)	59 (74.7 %)	0.918
• Private school	56 (25.7 %)	20 (25.3 %)	
Residence location			
• Inside Al-Ahsa	180 (82.6 %)	59 (74.7 %)	0.130
• Outside Al Ahsa	38 (17.4 %)	20 (25.3 %)	
Type of cellphone used for communication at the light out^a^			
• Receiving text messages	186 (85.3 %)	67 (84.8 %)	0.913
• Sending text messages	137 (62.8 %)	54 (68.4 %)	0.381
• Receiving phone calls	66 (30.3 %)	26 (32.9 %)	0.664
• Calling	47 (21.6 %)	14 (17.7 %)	0.469
During the past month, the number of times used			
• Every night	150 (68.8 %)	50 (63.3 %)	0.370
• Not every night	68 (31.2 %)	29 (36.7 %)	
Symptoms that affect the quality of personal or school life^a^			
• Headache	131 (60.1 %)	19 (24.1 %)	**<0.001 ∗∗**
• Insomnia	127 (58.3 %)	12 (15.2 %)	**<0.001 ∗∗**
• Losing energy	135 (61.9 %)	24 (30.4 %)	**<0.001 ∗∗**
• Distractibility	104 (47.7 %)	19 (24.1 %)	**<0.001 ∗∗**
• Tiredness	149 (68.3 %)	27 (34.2 %)	**<0.001 ∗∗**

∗∗ Significant at p < 0.05 level.

a Variable with multiple response answers.

b P-value has been calculated using Chi-square test.

When conducting a multivariate regression analysis (Table 4), it was revealed students who experienced headaches were predicted to increase the risk of having poor sleep quality by at least 2.22 times higher (AOR = 2.222; 95 % CI = 1.076–4.587; p = 0.031), while students who experienced insomnia had 4.62 increased risk for poor sleep quality (AOR = 4.642; 95 % CI = 2.191–9.836; p < 0.001). No significant effects were observed between sleep disorders in relation to age, gender, and other related symptoms that affect the personal or school quality of life after adjustments to a regression model (p > 0.05).

**Table 4:** Multivariate regression analysis to determine the significant independent risk factors for poor sleep quality ^(n = 297)^.

Factor	AOR	95 % CI	P-value
Age group			
• 15–16 years	Ref		
• 17–18 years	1.679	0.923–3.052	0.089
Gender			
• Male	Ref		
• Female	1.060	0.546–2.059	0.863
Symptoms that affect the quality of personal or school life a			
• Headache	2.222	1.076–4.587	**0.031 ∗∗**
• Insomnia	4.642	2.191–9.836	**<0.001 ∗∗**
• Losing energy	1.202	0.563–2.566	0.635
• Distractibility	0.973	0.455–2.083	0.944
• Tiredness	1.614	0.767–3.397	0.207

AOR – Adjusted Odd Ratio; CI – Confidence Interval.

∗∗ Significant at p < 0.05 level.

a Variable with multiple response answers.

## 4 Discussion

This study evaluates the influence of cell phone use after light out on sleep quality, headache, tiredness, and distractibility among high school students in the Eastern Province of Saudi Arabia. Based on the PSQI questionnaire, a high prevalence of poor sleep quality was detected among high school students (73.4 %). Among PSQI domains, sleep latency appeared to be the highest form of sleep disorder (mean score: 1.44), followed by daytime dysfunction (mean score: 1.38) and sleep medication (mean score: 1.29), while sleep efficiency showed the lowest rating (mean score: 1.04). Consistent with our findings, Akçay & Akçay (2018) reported that poor sleep quality was identified in 65.5 % of the adolescents. In addition, they discovered that 79.5 % of adolescents with poor sleep quality postponed their bedtime, and choosing to continue using mobile phones [14]. However, a study done by Alahdal et al. (2023) documented conflicting results on PSQI domains, wherein sleep disturbance and sleep medication showed the highest ratings, followed by sleep efficiency and sleep latency, with a global PSQI mean score of 6.63 out of 21 points [11]. The high prevalence of poor sleep quality among students is evidently seen in this study. Therefore, parents should make extra efforts to limit their children's screen time. Removing gadgets in children's rooms before bedtime can make a difference in their sleep quality.

The older age group (age 17–18 years) and female gender who were cell phone users at bedtime were more likely to complain of sleep disorders than the other high school students. This contradicted the reports of Pirdehghan et al. (2021) [7]. The use of Social Media at bedtime among boys with poor sleep quality was significantly higher than in girls. Also, the average duration of Social Media use has a direct link with depression. However, a study by Lee et al. (2023) [6] suggests that the adolescents who carried their cell phones to bed had poorer sleep quality than those without phones. Social Media use had no direct link with anxiety after controlling for confounders such as age and gender. Notwithstanding these reports, Tamura et al. (2017) [15] documented that the use of mobile phones for more than 5 h per day was concomitant with shorter sleep duration, but depression may not. However, mobile phone use of 2 h or more per day for social network services and online chats contributed to an increased risk of depression.

This study highlights the strong association between poor sleep quality and the symptoms that affected personal or school life. In particular, headache, insomnia, loss of energy, distractibility, and tiredness were prevalent in patients with poor sleep quality. However, in our predictive model, an increased risk of at least 2.22 and 4.64-fold higher was seen in the symptoms of headache and insomnia. Prior reports also suggest that poor sleep quality increases the risk of patients with migraine and Tension-type headaches as well as the frequency and severity in patients with migraine [16]. Hence, physicians should make extra efforts to improve the sleep of patients who are experiencing headaches to achieve better health outcomes.

This is in agreement with the previous reports done in Australia [17]. Tiredness made a substantial contribution to Depression Anxiety and Stress Scale (DASS) scores, with increasing levels of tiredness associated with increasing levels of depression, anxiety, and stress. This is corroborated by the study done in Iran [18]. A significant correlation occurred between using cell phones late at night and insomnia, tiredness, low energy, and headache, but not with distractibility. Insomnia was the only variable that remained significant after excluding stressful events. Not opposing these reports, a study conducted among American young adults [19] found that undergraduate students who used cell phones prior to sleep and read emotionally charged content before bed were likelier to report trouble sleeping. Findings suggest supporting Arousal theories and Sleep Displacement disruption sleep and delivering more perspectives into potential mechanisms for sleep disturbance among the young. There is ample evidence that supports the effect of cell phone use on sleep and psychological disorders. Measures must taken into consideration to alleviate this effect, such as engaging in some physical activities to mitigate the adverse effects of smartphone use at bedtime and improve the youth's sleep quality [20].

Notably, nearly seventy percent of our population regularly used cell phones every night, and only 2.4 % were non-cell phone users at bedtime. This mirrored the results of Pirdehghan et al. (2021) [7]. Nearly two-thirds (62.3 %) of students kept their cell phones in their bedrooms during sleep, which was consistent with the study by Zarghami et al. (2015) [18]. In contrast, Munezawa et al. (2011) [21] disclosed that only 17.6 % and 8.3 % used mobile phones regularly to send messages and calls after lights were out. The increasing use of cell phones at bedtime significantly contributes to sleep disturbance [14]. Thus, parents should prohibit their children from using any gadgets at bedtime to improve their sleeping patterns and prevent them from developing any symptoms associated with smartphone addiction. One key limitations of this study is the potential for selection bias due to the reliance on parents reporting symptoms related to their childrens’ sleep patterns and cell phone usage. The fact that the study’s required sample size have not completely met is also a notable limitations. Additionally, the study's cross-sectional design limits our ability to draw causal conclusions.

## 5 Conclusion

There was a high prevalence of poor sleep quality among high school students cell phone users after light out. Poor sleep varied by age and gender but not by students' location or school sector. The outcome of this study supports the literature that high school students' use of cell phones for messaging and calling after light out was associated with several forms of sleep disorders. Also, it increases the symptoms that affect personal or school life, particularly headaches and insomnia. The amount of time high school students spend using cell phones is a point of concern. Excessive use of cell phones can affect adolescent health both physically and psychologically. Hence, parents must control their children's intended use of gadgets by setting the bar on screen time, which could result in a better quality of personal or school life among them. Future longitudinal studies are needed to establish causal links better and track changes over time.

## CRediT authorship contribution statement

**Hanan Alrubaia:** Writing – original draft, Formal analysis, Data curation, Conceptualization. **Zainab Alabdi:** Writing – original draft, Visualization, Supervision, Formal analysis, Data curation. **Mohammed Alnaim:** Writing – original draft, Resources, Methodology, Formal analysis. **Nurah Alkhteeb:** Writing – original draft, Methodology, Data curation, Conceptualization. **Abdulmohsen Almulhim:** Writing – original draft, Formal analysis, Conceptualization. **Ammar Alsalem:** Writing – original draft, Methodology, Formal analysis. **Rimas Aldarwish:** Writing – original draft, Methodology, Conceptualization. **Ibtisam Algouf:** Writing – original draft, Formal analysis, Conceptualization. **Abdullah Almaqhawi:** Writing – review & editing, Writing – original draft, Supervision, Investigation.

## Ethics statement

Ethical approval of the study proposal from the Research Ethics Committee at 000000 (KFU-REC-2023-MAR-ETHICS691). Given the cross-sectional nature of the study and the self-administered questionnaire used, verbal informed consent was obtained from all participants.

## Data availability statement

The datasets used and analyzed during the current study are available from the corresponding author on reasonable request.

## Funding

No fund was received from any governmental or private Institutions.

## Declaration of competing interest

The authors declare the following financial interests/personal relationships which may be considered as potential competing interests:Abdullah Almaqhawi reports was provided by King Faisal University College of Medicine. Abdullah Almaqhawi reports a relationship with King Faisal University that includes: funding grants. The authors declare that they have no known competing financial interests or personal relationships that could have appeared to influence the work reported in this paper. If there are other authors, they declare that they have no known competing financial interests or personal relationships that could have appeared to influence the work reported in this paper.
