# Supplementary material for: The impact of cell phone use after light out on sleep quality, headache, tiredness, and distractibility among high school students: Cross sectional study
Source: Heliyon. 2025 Feb 14;11(4):e42655. doi: 10.1016/j.heliyon.2025.e42655 (PMC11908584; doi:10.1016/j.heliyon.2025.e42655)
Supplement: Multimedia component 1 [file mmc1.docx]

| **Question** | | | | **Response** | |
| --- | --- | --- | --- | --- | --- |
| **Biographical data and perception** | | | | | |
| - This questionnaire is intended for high school students | | | | | |
| 1. Gender | | | | - Male - Female | |
| 2. Age | | | | _________ | |
| 3. School sector | | | | - Public School - Private School | |
| 4. Where are you from ? | | | | - Al-Ahssa - Dammam - Alkhuboer - Al-Qateef - Jubail | |
| Table1: Frequency of Using Cell Phones After Lights are Out by Students | | | | | |
| Type of Use | Every Night | More Than Once a Week | Once a Week | 1 - 3 Times a Month | Never |
| Receiving text messages |  |  |  |  |  |
| Sending text messages |  |  |  |  |  |
| Receiving phone calls |  |  |  |  |  |
| Calling |  |  |  |  |  |

Supplemental material:

| Table2 : **The Relationship Between Using Cell Phones After Lights are Out and Headache, Insomnia, Losing Energy, Tiredness, and**  **Distractibility in Students** | |
| --- | --- |
| **In the past month, I have had:**  **(select all that apply)** | **This symptom has significantly affected the quality of my personal and/or school life** |
| Headache | \| Strongly disagree \| Disagree \| Neutral \| Agree \| Strongly agree \| \| --- \| --- \| --- \| --- \| --- \| \| Strongly disagree \| Disagree \| Neutral \| Agree \| Strongly agree \| \| Strongly disagree \| Disagree \| Neutral \| Agree \| Strongly agree \| \| Strongly disagree \| Disagree \| Neutral \| Agree \| Strongly agree \| \| Strongly disagree \| Disagree \| Neutral \| Agree \| Strongly agree \| |
| Insomnia |  |
| Losing Energy |  |
| Tiredness |  |
| Distractibility |  |

**Pittsburgh Sleep Quality Index (PSQI)**

Instructions: The following questions relate to your usual sleep habits during the past month only. Your answers should indicate the most accurate reply for the majority of days and nights in the past month. **Please answer all questions.**

1. During the past month, what time have you usually gone to bed at night?
2. During the past month, how long (in minutes) has it usually taken you to fall asleep each night?
3. During the past month, what time have you usually gotten up in the morning?
4. During the past month, how many hours of actual sleep did you get at night? (This may be different than the number of hours you spent in bed.)

| 5. During the past month, how often have you had trouble sleeping because you… | Not during the past month | Less than once a week | Once or twice a week | Three or more times a week |
| --- | --- | --- | --- | --- |
| a. Cannot get to sleep within 30 minutes |  |  |  |  |
| b. Wake up in the middle of the night or early morning |  |  |  |  |
| c. Have to get up to use the bathroom |  |  |  |  |
| d. Cannot breathe comfortably |  |  |  |  |
| e. Cough or snore loudly |  |  |  |  |
| f. Feel too cold |  |  |  |  |
| g. Feel too hot |  |  |  |  |
| h. Have bad dreams |  |  |  |  |
| i. Have pain |  |  |  |  |
| j. Other reason(s), please describe: |  |  |  |  |
| 6. During the past month, how often have you taken medicine to help you sleep (prescribed or  “over the counter”)? |  |  |  |  |
| 7. During the past month, how often have you had trouble staying awake while driving, eating meals, or engaging in social activity? |  |  |  |  |
|  | No problem at all | Only a very slight problem | Somewhat of a problem | A very big problem |
| 8. During the past month, how much of a problem has it been for you to keep up enough enthusiasm to get things done? |  |  |  |  |
|  | Very good | Fairly good | Fairly bad | Very bad |
| 9. During the past month, how would you rate your sleep quality overall? |  |  |  |  |

|  | No bed partner or  room mate | Partner/roommate in  other room | Partner in same room but  not same bed | Partner in same bed |
| --- | --- | --- | --- | --- |
| 10. Do you have a bed partner or roommate |  |  |  |  |
|  | Not during  the past month | Less than once a week | Once or twice a week | Three or  more times a week |
| If you have a room mate or bed partner, ask him/her how often in the past month you have  had: |  |  |  |  |
| a. Loud snoring |  |  |  |  |
| b. Long pauses between breaths while asleep |  |  |  |  |
| c. Legs twitching or jerking while you sleep |  |  |  |  |
| d. Episodes of disorientation or confusion  during sleep |  |  |  |  |
| e. Other restlessness while you sleep, please describe: |  |  |  |  |

References :

1. Buysse, DJ, Reynolds CF, Monk TH, Berman SR, Kupfer DJ: The Pittsburgh Sleep Quality Index (PSQI): A new instrument for psychiatric research and practice. Psychiatry Research 28:193-213, 1989
2. Langeveld, J. H., Koot, H. M., & Passchier, J. (1997). Headache intensity and quality of life in adolescents. How are changes in headache intensity in adolescents related to changes in experienced quality of life?. Headache: The Journal of Head and Face Pain, 37(1), 37-42.
3. Zarghami, M., Khalilian, A., Setareh, J., & Salehpour, G. (2015). The impact of using cell phones after light-out on sleep quality, headache, tiredness, and distractibility among students of a university in North of Iran. Iranian journal of psychiatry and behavioral sciences, 9(4).‏
4. Suleiman KH, Yates BC, Berger AM, Pozehl B, Meza J. Translating the Pittsburgh Sleep Quality Index into Arabic. Western Journal of Nursing Research. 2009;32(2):250–68. doi:10.1177/0193945909348230
